# Targeted Delivery of Cisplatin-Derived Nanoprecursors via a Biomimetic Yeast Microcapsule for Tumor Therapy by the Oral Route

**DOI:** 10.7150/thno.35353

**Published:** 2019-08-21

**Authors:** Xing Zhou, Kaijian Ling, Mengyu Liu, Xiangjun Zhang, Jun Ding, Yan Dong, Zhiqing Liang, Jianjun Li, Jianxiang Zhang

**Affiliations:** 1 Department of Pharmaceutics, College of Pharmacy, Third Military Medical University, Chongqing 400038, China; 2 Department of Obstetrics and Gynaecology, Southwest Hospital, Third Military Medical University, Chongqing 400038, China; 3 Department of Ultrasound, Southwest Hospital, Third Military Medical University, Chongqing 400038, China; 4 Department of Oncology and Southwest Cancer Center, Southwest Hospital, Third Military Medical University, Chongqing 400038, China

**Keywords:** cisplatin, nanoprecursor, yeast microcapsule, oral delivery, targeted tumor therapy

## Abstract

Targeted therapy via the patient-friendly oral route remains the holy grail of chemotherapy for cancer. Herein we report a yeast-derived platform for targeted oral delivery of cisplatin (CDDP) that is one of the most effective drugs for chemotherapy of various types of cancers.

**Methods:** The optimal conditions were first established to fabricate yeast microcapsules (YCs) with desirable loading capability. Then, CDDP-derived precursor nanoparticles (PreCDDP) were prepared and packaged into YC to produce orally deliverable PreCDDP/YC. The physiochemical properties, *in vitro* drug release profiles, *in vitro* antitumor activity, oral targeting capability, *in vivo* pharmacokinetics, and *in vivo* efficacy of the YC-based biomimetic delivery system were examined.

**Results:** YCs obtained under the optimized condition showed desirable loading efficiency for quantum dots that were used as a model nanocargo. *In vitro* experiments demonstrated rapid endocytosis and prolonged retention of YC in macrophages. By electrostatic force-mediated self-deposition, PreCDDP was efficiently loaded into YC. PreCDDP/YC showed potent cytotoxicity in different tumor cells, indicating that PreCDDP loaded in YC maintained its antitumor activity after intracellular release. As compared to CDDP and PreCDDP, orally administered PreCDDP/YC displayed significantly higher bioavailability. Post oral delivery, YC could accumulate in A549 human lung carcinoma xenografts in mice, achieving by monocyte/macrophage-mediated translocation via the lymphatic system. Through this targeting effect, orally administered PreCDDP/YC showed desirable efficacy in A549 xenograft-bearing mice, which was comparable to that of free CDDP administered by intravenous injection. Orally administered free CDDP, however, did not afford antitumor effects. Furthermore, oral treatment with PreCDDP/YC displayed better safety than free CDDP administered via the oral or intravenous route.

**Conclusions:** This biomimetic approach can serve as an effective strategy to develop targeted oral chemotherapies based on CDDP or its derivatives.

## Introduction

Platinum-based anticancer agents, including cisplatin, carboplatin, and oxaliplatin, are approved worldwide for treating a number of cancers in humans 1. Cisplatin, *i.e.* cis-diamminedichloroplatinum (CDDP) approved in 1978 by FDA, is one of the most effective drugs for chemotherapy of various types of cancers such as lung cancer, ovarian cancer, cervical cancer, and breast cancer 2. Generally, CDDP is administered intravenously (*i.v.*) as short-term infusion in saline for the treatment of solid tumors. Unfortunately, *i.v.* administration of CDDP frequently leads to side effects like nephrotoxicity and neurotoxicity as well as toxicity to the gastrointestinal tract 3-7.

To minimize its side effects and further improve its efficacy, CDDP has been formulated into a large number of nanotherapies 1, 8-13. In this context, a diverse array of nanomaterials have been designed to deliver CDDP or its prodrugs, including carbon nanotubes 14, gold nanorods 15, inorganic nanoparticles 16, metal-organic frameworks 17, liposomes 18, lipid nanoparticles 19, nanogels 20, nanocomplexes 21, polymeric micelles 22, 23, polymer nanoparticles 24, 25, CDDP-linked polymeric prodrugs 26, 27, hybrid nanoparticles 28, and other supramolecular nanostructures 29, 30. Thus engineered platinum nanotherapies can be passively targeted to tumor sites via the enhanced permeability and retention (EPR) effect 31. Also, tumor targeting capacity may be further enhanced by decorating CDDP-loaded nanoparticles with different targeting moieties 24, 32-35. Unambiguously, these extensive and intensive studies have made great achievements. In particular, several CDDP nanotherapies derived from polymeric micelles or liposomes have been advanced to clinical trials 22, 36, 37. Nevertheless, challenges remain in the development of efficacious, safe, and translational CDDP nanotherapies for targeted tumor therapy. For the majority of currently developed CDDP nanoformulations that need to be administered via the *i.v.* route, their targeting capability is dominated by the EPR effect 31. However, delivery efficiency of this targeting strategy can be notably attenuated by different pathophysiological hurdles, such as unexpected surface coating of biomolecules in the blood 38, clearance by the host mononuclear phagocytic system 39, poor and/or abnormal microvasculature in tumors 40, limited penetration in poorly permeable tumors 41, and high interstitial fluid pressure in solid tumors 42. These factors may have mainly contributed to the low delivery efficiency of nanoparticles at tumor sites 43. In addition, *i.v.* administration of cancer nanomedicines needs strict regulations due to safety concerns. Furthermore, the invasive *i.v.* injection more often causes poor patient compliance. Accordingly, other innovative approaches are desperately required to develop more effective, safe, and patient-friendly nanotherapies for cancer patients 44.

On the other hand, oral drug delivery is preferred for the treatment of different diseases, due to its multiple advantages such as convenience, relatively good safety profile, high patient compliance and adherence, and desirable cost-effectiveness 45, 46. Oral administration of anticancer therapeutics may considerably change current treatment modalities of chemotherapy and greatly improve the quality of life of patients with cancers 47. In addition, there is also a potential significant economic advantage of switching cancer treatment from inpatient to outpatient chemotherapy 48. During the past decades, there is a growing interest in developing nanomedicines for oral chemotherapy 47, 49, 50. In the case of platinum-based anticancer drugs, generally used CDDP, carboplatin, and oxaliplatin have been found ineffective after oral administration at normal doses, largely resulting from their low solubility and poor bioavailability in the gastrointestinal tract 2, 51. One extensively investigated strategy is to discover orally active platinum drugs 1, 2, 52. Unfortunately, despite extensive studies over a decade, no new platinum compounds have received worldwide approval. Alternatively, the micro- or nanoparticle-based delivery approach can be employed to realize oral chemotherapy based on CDDP or its derivatives. However, in contrast to other anticancer agents, extremely limited studies are available with respect to oral delivery of platinum drugs for targeted tumor therapy 53. Currently, development of orally accessible delivery systems that can efficiently promote absorption, enhance tumor targeting, and reduce toxicity of platinum drug is still an intriguing topic of drug discovery.

Increasing evidence has demonstrated that the biomimetic approach is highly promising for developing effective therapies for the treatment of diverse diseases by the oral route 54-58, varying from inflammation resolution to tumor therapy. In these cases, bacteria and yeast cells as well as their bioengineered products were investigated as either therapeutics or delivery carriers 59-61. Most recently, we found that the yeast-derived capsule (YC) can efficiently package different positively charged nanoparticles including different nanoprobes and nanotherapies 62-64. After oral administration, nanoparticles loaded in YC can accumulate in distant diseased sites of acute and chronic inflammation, thereby affording beneficial therapeutic effects in different models of inflammatory diseases. Also, orally administered YCs may be transported to tumor sites in mice bearing murine B16F10 melanoma or MCF-7 human breast cancer xenografts as well as in rats with Walker 256 carcinoma 62. On the basis of these promising findings and with the aim to develop an effective and safe delivery system for oral administration of platinum drugs, herein we employed a nanoprecursor-packaging strategy, in which CDDP was first transformed to a water-soluble prodrug PreCDDP that can form nanoparticles in aqueous solution. Subsequently, PreCDDP nanoparticles were loaded into YC to afford an orally targeted therapy. In addition to physicochemical characterization and *in vitro* cellular evaluations, *in vivo* studies were performed in mice with A549 bronchioloalveolar carcinoma xenografts to demonstrate the targeting capability and therapeutic effect.

## Materials and methods

### Materials

Yeast of *Saccharomyces cerevisiae* was purchased from Lesaffre International Corporation (France). Positively coated CdSe/ZnS quantum dots (QDs) with an emission wavelength at 620 nm (QD620) were obtained from Ocean NanoTech, LLC (U.S.A.). Branched polyethyleneimine (PEI) with molecular weight (*M*_w_) of 25 kDa was purchased from Sigma (U.S.A.). Cyanine 7.5 NHS ester (Cy7.5) and Cyanine 5 NHS ester (Cy5) were supplied by Lumiprobe (U.S.A.). 4', 6-Diamidino-2-phenylindole (DAPI) was purchased from Invitrogen (U.S.A.). Cisplatin, *i.e.* cis-diamineplatinum (II) dichloride (CDDP) was purchased from Thermo Fisher (U.S.A.). Penicillin, streptomycin, and fetal bovine serum (FBS) were purchased from Gibco (U.S.A.). RPMI 1640 medium was obtained from HyClone (U.S.A.). All the other reagents are commercially available and used as received.

### Fabrication of yeast capsules under different conditions

Yeast capsules (YCs) were fabricated according to the previously reported method with minor modification 62. First, the optimal time for alkali treatment was examined. To this end, 45 g yeast was suspended in 450 mL of 1 M NaOH, and the obtained suspension was heated at 80°C. At 0.5, 1, 1.5, 2.0, 4.0, 6.0, 8.0, or 12 h, 50 mL of suspension was withdrawn. The samples were collected by centrifugation at 2500*g* for 5 min, and rinsed twice with 50 mL deionized water. Subsequently, the samples were separately dispersed in aqueous solution with pH 4.5 and incubated at 55°C. After 1 h, the yeast samples were collected after centrifugation at 2500 *g* for 5 min and thorough washing with 50 mL of deionized water. Thus obtained samples were washed with 10 mL of isopropyl alcohol four times. After additional rinsing with 10 mL of acetone twice, the collected yeast samples were dried under vacuum.

In a separate cohort of experiments, time of the acid treatment was optimized. In this case, 45 g yeast was suspended in 450 mL of 1 M NaOH at 80°C. After 1 h, the suspension was centrifuged at 2500*g* for 5 min, and rinsed twice with 450 mL of deionized water. The obtained sample was dispersed in aqueous solution at pH 4.5 and incubated at 55°C. At the time point of 0, 0.5, 1, 1.5, 2, 3, 4, or 6 h, 50 mL of suspension was withdrawn, centrifuged at 2500*g* for 5 min, and thoroughly washed with 50 mL of deionized water. The corresponding samples were separately washed with 10 mL of isopropyl alcohol four times, followed by rinsing with 10 mL of acetone twice. Finally, the harvested yeast samples were dried under vacuum.

### Preparation of QD620 containing yeast capsules

YCs obtained under different processing conditions were first incubated in 100 μL of carbonate buffer saline (CBS) at pH 9.2 at 37°C for 2 h. Then QD620 was added at 24 nmol QD per mg YC and fully mixed. After overnight incubation at 37°C, QD620-loaded YCs (QD620/YCs) were harvested by centrifugation at 2500 *g* for 5 min. The obtained precipitate was rinsed with 2 mL of deionized and centrifuged at 2500 *g* for 5 min to collect both QD620/YC samples and supernatant. The unloaded QD620 in the combined supernatant was determined by fluorescence spectrometry (Hitachi, F7000). Finally, QD620/YC was harvested after lyophilization.

### Preparation of yeast capsules labeled with Cy7.5 or Cy5

To synthesize Cy7.5-conjugated PEI (Cy7.5-PEI), 6 mg Cy7.5 NHS ester in 2 mL of DMSO was added into 2 mL of DMSO containing 64 mg PEI, the obtained solution was stirred in the dark at 40°C for 12 h. After complete reaction, Cy7.5 nanoparticles (Cy7.5 NPs) were obtained by dialysis of the reaction solution against deionized water for 24 h. Cy5 nanoparticles (Cy5 NPs) were prepared by the similar procedures using Cy5-conjugated PEI (Cy5-PEI).

To fabricate Cy7.5 NP-loaded YC (Cy7.5 NP/YC), 60 mg YC was dispersed in 2 mL of CBS at pH 9.2 by sonication and incubated at 37°C for 1 h. Then, 600 μL of aqueous solution containing Cy7.5 NP was added, followed by incubation at 37°C for 1 h under the dark. Cy7.5 NP/YC was collected after centrifugation at 2500*g* for 5 min and rinsing with deionized water. Similarly, Cy5 NP/YC was prepared.

### Preparation of yeast capsules loaded with a CDDP nanoprecursor

A CDDP precursor (PreCDDP) was prepared according to literature 65. Briefly, 60.0 mg CDDP was dispersed into 1 mL of deionized water, into which 66.2 mg AgNO_3_ was added. The mixture was stirred magnetically and heated at 60°C for 3 h, which was then stirred in a flask protected from light with aluminum foil. After 12 h, thus obtained solution was centrifuged at 14000*g* for 15 min to remove precipitated AgCl. After the supernatant was filtered through a 0.22 μm syringe filter, it was freeze-dried to give rise to PreCDDP, *i.e.* cis-[Pt(NH_3_)_2_(H_2_O)_1_(Cl)_1_] (NO_3_)_1_.

To package PreCDDP into YC, 1 mL of aqueous solution containing 0.4 mg/mL PreCDDP was mixed with 1 mL of aqueous solution containing 2 mg/mL YC, and incubated at 37°C for different periods of time. Then the mixture was centrifuged at 2500 *g* for 5 min to collect PreCDDP-loaded YC (PreCDDP/YC), while the supernatant was also harvested to quantify unloaded PreCDDP by atomic absorption spectroscopy (AAS, FGA-7000A, Shimadzu). Similar procedures were followed to examine the effect of pH values of PreCDDP solution on its loading efficiency.

### Characterization of various particles

Dynamic light scattering (DLS) and ξ-potential measurements were performed on a Malvern Zetasizer Nano ZS instrument at 25°C. Transmission electron microscopy (TEM) observation was carried out on a Tecnai-10 microscope (Philips, the Netherlands) operating at an acceleration voltage of 80 kV. Scanning electron microscopy (SEM) was conducted on a FIB-SEM microscope (Crossbeam 340, Zeiss). Confocal laser scanning microscopy (CLSM) observation was performed using a Zeiss LSM510 laser scanning confocal microscope.

### *In vitro* release study

*In vitro* release experiments were performed in solutions simulating pH conditions in the gastrointestinal tract. To this end, 5 mg of PreCDDP/YC was dispersed into 1.5 mL of aqueous solution at pH 1.2 and incubated at 37°C. At predetermined time intervals, 0.5 mL of release medium was withdrawn after samples were centrifuged at 6000 *g* for 5 min, and fresh medium was supplemented. After 2 h, the release medium was switched into 0.01 M PBS at pH 7.4, followed by similar protocols. The CDDP concentrations in release buffers were quantified by AAS.

### Cell culture

RAW264.7 mouse macrophage, HeLa human cervical cancer cell, human hepatocellular carcinoma HepG2 cell, MCF-7 human breast cancer cell, and human A549 lung adenocarcinoma cells were obtained from the Cell Bank of the Committee on Type Culture Collection of Chinese Academy of Sciences (Shanghai, China). The multidrug resistance cell subline of MCF-7 (MCF-7/ADR) was derived from the parental cells by selection with doxorubicin 66. The cells were cultured in RPMI 1640 medium containing 10% FBS, 100 U/mL penicillin, and 100 μg/mL streptomycin. All cells were incubated at 37°C in a humidified atmosphere with 5% CO_2_ for 24 h before further experiments.

### Examination on cellular uptake of yeast capsules

To visualize YC internalization by RAW264.7 macrophages, cells were seeded onto glass coverslips placed in 6-well plates. After incubation with 2 mL of growth medium for 12 h, the cells were washed with PBS, replenished with 2 mL of fresh medium, and incubated with QD620/YC. After incubation for specified time periods, the cells were washed with PBS, fixed with 4% paraformaldehyde, and counterstained with DAPI. Fluorescence microscopy observation was performed by CLSM. In a separate study, RAW264.7 cells were incubated with QD620/YC for 2 h, rinsed with PBS, and subsequently observed at defined time points by CLSM after DAPI staining.

### Cytotoxicity evaluations in different cells

To evaluate cytotoxicity of YC, PreCDDP, or PreCDDP/YC in different cells, cells including RAW264.7, MCF-7, MCF-7/ADR, HepG2, HeLa, and A549 were seeded in 96-well plates at a density of 1 ×10^4^ cells per well in 100 μL of growth medium. After 24 h, the culture medium was replaced by 100 μL of fresh medium containing YC, PreCDDP, or PreCDDP/YC at various doses and incubated for 24 or 48 h. The cell viability was quantified by CCK-8 assay.

### Animals

All animal experiments were performed in accordance with the Guide for the Care and Use of Laboratory Animals proposed by National Institutes of Health. All the animal care and experimental protocols were performed under review and approval from the Animal Ethical and Experimental Committee of the Third Military Medical University (Chongqing, China). Male Sprague-Dawley rats (180-220 g), male BALB/c mice (20-25 g), and BALB/c athymic nude mice (18-22 g) were obtained from the Animal Center of the Third Military Medical University. Animals were housed in standard cages under conditions of optimum light, temperature, and humidity, with *ad libitum* access to water and food. Animals were acclimatized to the laboratory for at least 7 days before experiments.

### Tumor targeting after oral administration of Cy7.5-labeled YC in nude mice

To establish A549 xenografts, A549 cells were subcutaneously inoculated in nude mice (1 × 10^7^ cells in each mouse). When the tumors reached 1 cm^3^ in volume, the mice were randomly assigned into 3 groups (*n* = 4). Oral administration of 0.3 mL of suspension containing Cy7.5 NP or Cy7.5 NP/YC was then performed. In the control group, 0.3 mL of saline was orally administered. The fluorescence intensities at specified time points were measured by an IVIS Spectrum living imaging system (PerkinElmer, U.S.A.). Splitting of the emission signals of Cy7.5 was conducted to clearly show the fluorescent signals in the tumors. After 48 h, the mice were euthanized. Tumors and main organs were excised for further analysis. *Ex vivo* imaging was performed for the excised tumors.

In a separate experiment, Cy5 NP/YC was orally administered daily to nude mice bearing A549 xenografts for three sequential days. At 12 h after the last dosing, mice were euthanized. Peyer's patches, mesenteric lymph node, and tumors were harvested for further analysis. Cryosections of tumor tissues were prepared and immunofluorescence analysis was performed after staining with FITC-labeled CD68 antibody (Abcam) and DAPI.

### *In vivo* pharmacokinetic study

Twenty male Sprague-Dawley rats were randomly assigned into four groups. In one group, CDDP solution was *i.v.* injected at 6 mg/kg of CDDP. Other three groups were separately administered with CDDP, PreCDDP, or PreCDDP/YC by gastric gavage at a dose corresponding to 6 mg/kg of CDDP. All rats fasted overnight before administration. At predetermined time points after administration, blood samples were collected. In addition, peripheral blood monocytes were isolated by using a monocyte-separating medium. Then Pt concentrations in plasma and blood monocytes were quantified by high performance liquid chromatography (HPLC) 67. Representative pharmacokinetic parameters were analyzed by DAS 3.2 software.

### *In vivo* antitumor efficacy

The A549 xenograft model was established by inoculating A549 cells (1 × 10^7^) subcutaneously in the left axilla of nude mice. When the tumors reached 100-150 mm^3^ in volume, the mice were randomly assigned into different groups. Then, different formulations were administered by either oral gavage or *i.v.* injection at defined doses. The tumor size was measured by a caliper at specified time points. Tumor volume was calculated by the formula for an ellipsoid sphere (*a* × *b*^2^/2), where *a* and *b* refer to the major and minor axes of tumor, respectively. During treatment, changes in the body weight were monitored. After treatment, the mice were euthanized, and tumors were excised for further analysis. In addition, different major organs were isolated for histopathological examination based on sections stained with hematoxylin and eosin stain (H&E).

### Preliminary safety studies

Male BALB/c mice were randomly divided into 4 groups (n = 6). Mice were orally administered with saline, CDDP (6.0 mg/kg), or PreCDDP/YC (6.0 mg/kg) every three days. In another group, CDDP aqueous solution was administered by *i.v.* injection via the tail vein at 6.0 mg/kg of CDDP every three days. The body weight was examined during treatment. After 6 times of treatments, mice were euthanized. Blood samples were collected for hematological analysis and clinical biochemistry tests. Main organs were isolated and weighed for calculation of the organ index. For typical organs, H&E sections were prepared and analyzed.

### Complete blood counts and clinical chemistries

Blood samples were collected in EDTA spray-coated tubes and immediately analyzed (Sysmex KX-21, Sysmex Co., Japan). The plasma concentrations of alanine aminotransferase (ALT), blood urea (UREA), creatinine (CREA) were also quantified (Roche Cobas C501, Roche Co., Switzerland).

### Statistical analysis

All data are expressed as mean ± standard deviation (SD). The SPSS 18.0 statistical package was used for data analyses. After tests for data homogeneity, independent continuous variables were processed through a variance analysis (ANOVA). Statistical analyses were performed using the one-way ANOVA test with two-tailed Student's *t*-test for experiments consisting of more than two groups, and an unpaired t-test in experiments with two groups. For paired samples, statistical analysis was conducted using the paired samples *t*-test. Statistical significance was assessed at p < 0.05.

## Results and discussion

### Study design

**Figure 1:**
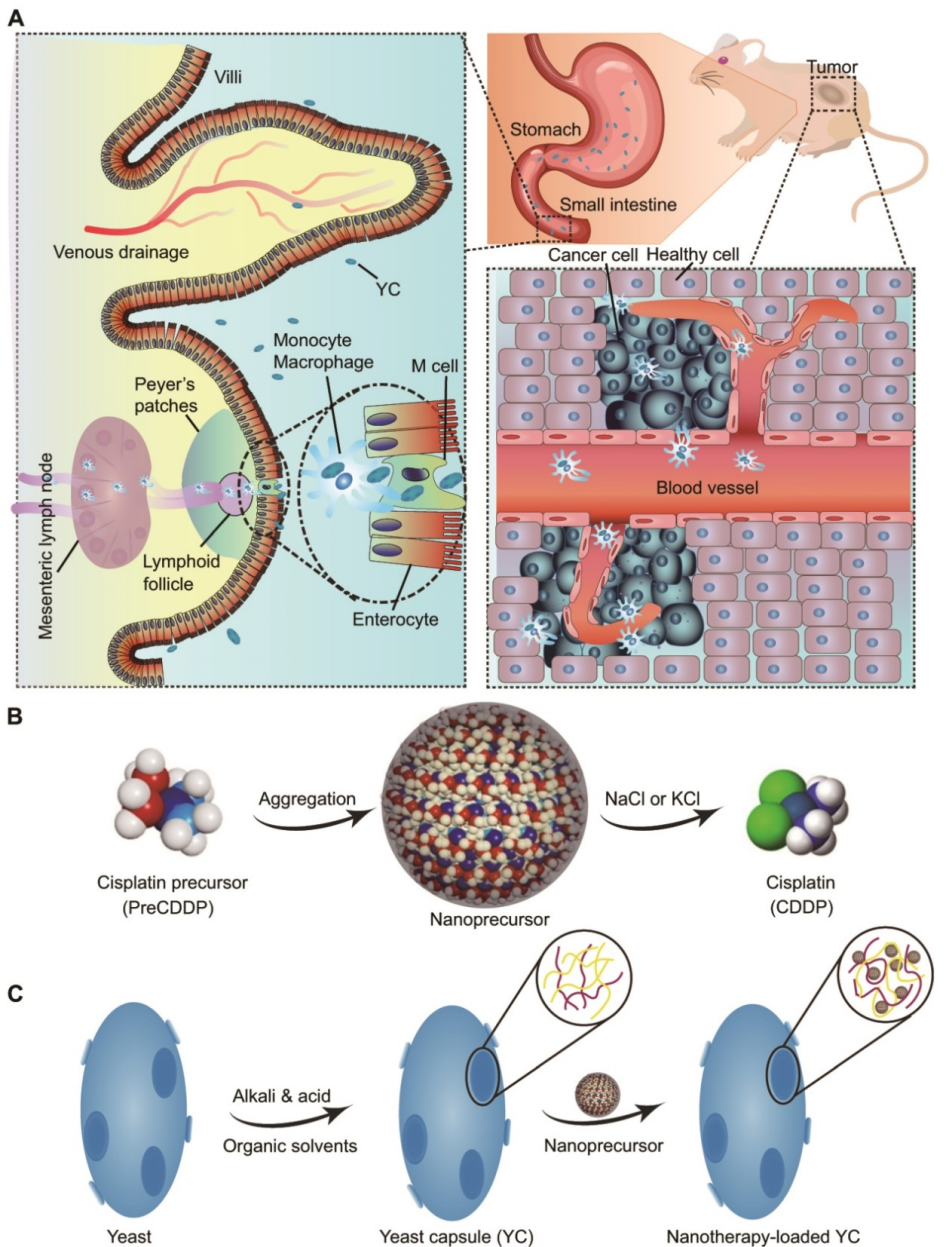
Schematic of yeast capsule-mediated oral delivery of a CDDP nanoprecursor for targeted tumor therapy. (A) Translocation and targeted tumor treatment via orally delivered yeast capsules (YCs) loaded with CDDP nanoprecursors. (B) Sketch of the formation and hydrolysis of a CDDP-derived PreCDDP nanoprecursor. (C) Loading of the nanoprecursor into YC.

We hypothesize that YC can function as an efficient biomimetic carrier for targeted delivery of platinum drugs via the oral route (Figure 1A). In this strategy, a platinum drug is first transformed into a precursor compound that can form nanoparticles via molecular aggregation (Figure 1B). Thus obtained nanoprecursors are further loaded into YC via electrostatic interactions (Figure 1C). On the one hand, this strategy can improve the loading content of platinum drugs in YC. On the other hand, packaging of nanoprecursors into YC is able to avoid side effects of pristine drugs, resulting from premature release and nonspecific distribution. As a proof of concept, CDDP was used as a candidate platinum drug (Figure 1B).

### Physicochemical properties and loading capability of yeast capsules

Whereas yeast-derived capsules were investigated for delivering therapeutics including nucleic acids, proteins, and nanotherapies in previous studies 58, 62, 68-71, the effect of their physicochemical characters on loading capacity remains elusive. Since the processing parameters largely dominate the characteristics of resulting YCs, we examined the effects of the time used for sequential alkali and acid treatments. After different time periods of treatment with 1 M NaOH, YCs were collected for characterization. We found no significant changes in average size (Figure S1A). All YCs harvested at different time points showed negative zeta-potential values (Figure S1B). Nevertheless, considerable differences could be observed from transmission electron microscopy (TEM) images (Figure S1C). As compared to intact yeast (Figure S2), the core content of yeast gradually decreased after treatment with NaOH for different times. However, additionally prolonged treatment may lead to large pores on resultant YCs, which is especially true in the case at 8 and 12 h.

Our previous studies demonstrated that positive nanoparticles (NPs) can be effectively packaged into YCs 62, 63. Therefore a positively charged quantum dot with emission at 620 nm (QD620) was employed as a fluorescent nanoprobe to explore the loading capacity of different YCs. Direct observation by confocal laser scanning microscopy (CLSM) revealed gradually enhanced red fluorescent signals in YCs collected at 0.5, 1, and 1.5 h (Figure S1D). However, fluorescent intensities of QD620 were notably decreased for YCs with NaOH treatment time of 2, 4, or 6 h. In addition, significant peripheral distribution of fluorescent signals were observed for YCs obtained after prolonged treatment in alkali solution. This finding was further affirmed by quantification via fluorescence spectroscopy (Figure S1E-F), which revealed that both loading contents and loading efficiency of QD620 followed a parabola profile, with the maximal value for YC at 1.5 h.

Then we interrogated the possible effects of time required for the acid treatment. It can be found that YCs collected after varied times of treatment at pH 4-5 showed comparable particles size (Figure S3A). In addition, YCs obtained under these different conditions displayed negative zeta-potential values (Figure S3B). Observation by TEM indicated the gradually decreased core content for YCs treated with a mildly acidic buffer for different time periods (Figure S3C). Notably, YC without treatment at pH 4-5 showed the highest core content as illustrated by the typical TEM image, suggesting this treatment is critical for effective removal of core materials. Of note, large pores were formed in YCs with long treatment time at pH 4.5. In line with this result, CLSM observation revealed relatively low loading of QD620 in YC without acidic treatment (Figure S3D), while high loading was observed for YC at 0.5 h. Quantification of both loading efficiency and loading contents by fluorescence measurement also confirmed this result (Figure S3E-F). Accordingly, based on these results, 1.5 h of NaOH treatment followed by 0.5 h of treatment at pH 4.5 was considered to be the optimal treatment procedure. YC derived from this condition showed desirable loading capability, in which the loading content of QD620 can be directly enhanced by increasing its feeding (Figure S4).

### Preparation and characterization of YCs loaded with a CDDP nanoprecursor

**Figure 2:**
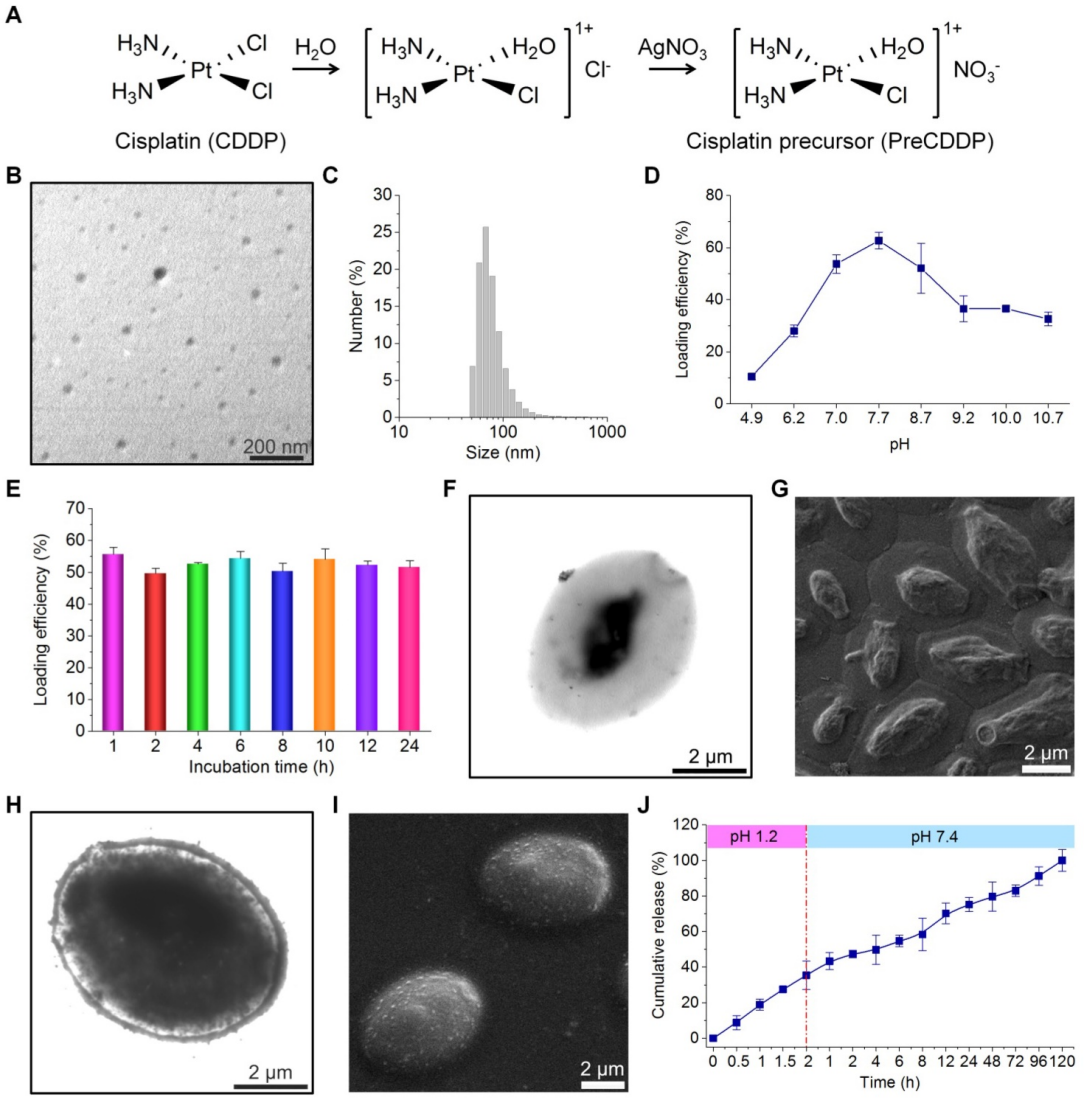
Fabrication and characterization of PreCDDP-loaded YCs. (A) The synthetic route of a CDDP precursor (PreCDDP). (B-C) TEM image (B) and size distribution profile (C) of PreCDDP-derived nanoprecursors in aqueous solution. (D-E) The effect of buffer pH values (D) and incubation time (E) on loading efficiency of PreCDDP in YC. (F-G) TEM (F) and SEM (G) images of YC prepared under optimized core-removing conditions. (H-I) TEM (H) and SEM (I) images of PreCDDP-loaded YC. (J) *In vitro* release profile of PreCDDP/YC in aqueous solutions simulating gastrointestinal conditions. Data in (D, E, J) are mean ± SD (n = 3).

Due to its low solubility in water (2.5 mg/mL at 25°C), CDDP cannot be efficiently packaged into YCs. Accordingly, a water soluble CDDP precursor (PreCDDP) was firstly prepared according to a previously established method with minor revisions (Figure 2A) 65. Interestingly, observation by TEM and measurement by dynamic light scattering (DLS) indicated PreCDDP formed nanoscale aggregates in aqueous solution (Figure 2B-C), with mean size of 82 nm and zeta-potential of +2.7 mV. It should be noted that YC obtained under the conditions of 1.5 h of NaOH treatment followed by 0.5 h of treatment at pH 4.5 was used for PreCDDP loading and subsequent studies.

To optimize entrapment efficiency of PreCDDP in YC, the exact loading process was examined. Since the pH value of buffer solutions may affect the swelling degree of YC 62, 68, its effect on PreCDDP loading was interrogated. Initially, the loading efficiency remarkably increased from 10.5% to 28.0%, 53.8%, and 62.7% as the buffer varied from pH 4.9 to pH 6.2, pH 7.0, and pH 7.7, respectively (Figure 2D). Nevertheless, further increase in buffer pH reduced PreCDDP loading. Based on this result, PBS with pH 7.7 was found desirable for effective PreCDDP packaging in YC. This should be related to a balance of PreCDDP diffusion into or out of YC and its retention under various pH conditions. On the other hand, incubation time periods ranging from 1 to 24 h showed negligible effects on PreCDDP entrapment efficiency (Figure 2E).

In the case of blank YCs, they were partially filled in the core (Figure 2F), exhibiting collapsed structure after drying (Figure 2G). By contrast, the interior of YC was largely filled post loading, as clearly illustrated by TEM image (Figure 2H). PreCDDP/YC displayed plump-like morphology even after drying (Figure 2I), reminiscent of intact yeast (Figure S5). In view of the presence of small protrusions on the YC surface, PreCDDP should be loaded in the interior of YC and simultaneously absorbed on the YC wall. On the one hand, electrostatic forces between PreCDDP nanoparticles and YC would account for effective PreCDDP loading, agreeing with our previous studies on other positive nanoparticles 62, 63. In addition, coordination interactions between Pt and residual components (such as the fragments of proteins and nucleic acids) in YCs may have contributed to effective loading of PreCDDP in YC.

Subsequently, *in vitro* release study was performed in solutions simulating gastrointestinal pH conditions. It was found that independent of media with varied pH values, CDDP or other forms of PreCDDP could be gradually released from PreCDDP/YC (Figure 2J). This result indicated that PreCDDP encased in YC can be efficiently released in aqueous solutions. Of note, PreCDDP/YC was incubated in an excessive amount of aqueous solutions for *in vitro* release tests, which may remarkably accelerate the dissolution of CDDP. In the real situation of the gastrointestinal tract, it is not filled with large volumes of liquid media, but contains a considerable amount of solid and semi-solid substances. Therefore, the *in vitro* release profile of PreCDDP/YC in the media simulating pH conditions of the gastrointestinal tract does not indicate that preCDDP/YC will be released prematurely. In addition, although relatively rapid release was observed in the first 6 h, release of the remained drug (~50%) sustained for about 110 h. On the other hand, release of PreCDDP/YC at pH 1.2 suggested that its stability should be further improved by optimizing formulations to avoid premature release in the gastric environment.

### Cellular uptake and *in vitro* biological effects in different cells

**Figure 3:**
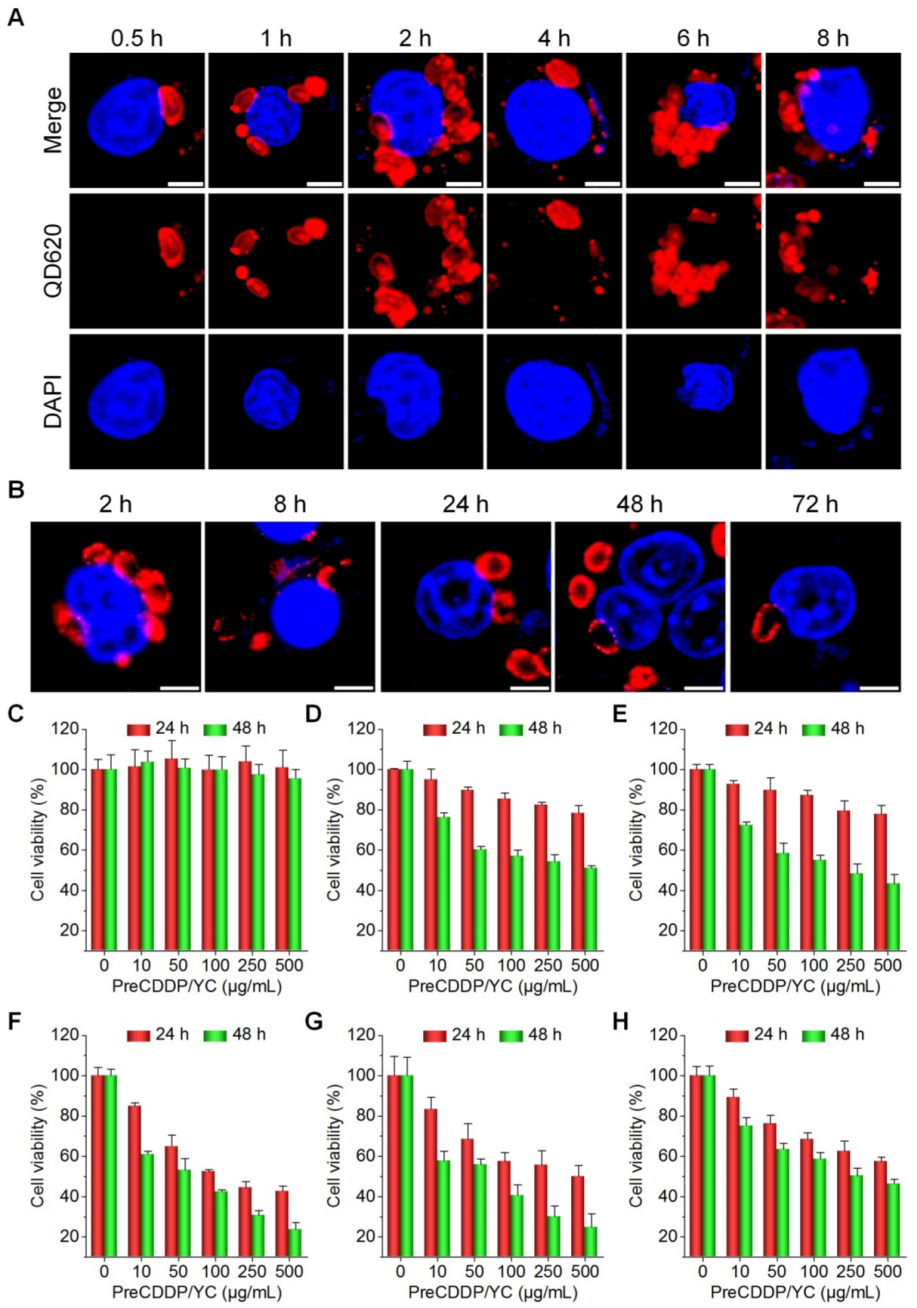
Cellular uptake and *in vitro* biological effects in different cells. (A) Time-dependent internalization of QD620/YC in RAW264.7 cells. (B) Cellular retention of QD620/YC in RAW264.7 cells. (C) Cell viability of RAW264.7 cells after incubation with various doses of PreCDDP/YC for 24 or 48 h. (D-H) Cytotoxicity of PreCDDP/YC in different tumor cells after incubation for 24 or 48 h, including HepG2 hepatocellular carcinoma cell (D), HeLa human cervical cancer cell (E), A549 human lung carcinoma cell (F), MCF-7 breast cancer cell (G), and multidrug resistant MCF-7 cell (MCF-7/ADR) (H). For all cell viability experiments, the indicated concentrations represent the doses of PreCDDP/YC. Scale bars in (A-B) represent 5 μm. Data are mean ± SD (n = 5).

After oral administration, YCs were dominantly delivered via macrophages to realize their targeting capability in different inflammatory diseases 58, 62, 63. Using QD620-labeled YC (QD620/YC), we investigated cellular uptake behaviors of YC in murine RAW264.7 macrophages. Even after 0.5 h of incubation, significant fluorescent signals of QD620 could be observed in RAW264.7 cells (Figure 3A). With prolonged incubation, fluorescent intensities in macrophages dramatically increased. In addition, intracellular distribution of internalized QD620/YC could be observed at 72 h after incubation (Figure 3B). Accordingly, endocytosed YC can be maintained in macrophages for a relative long period of time.

Then the possible effect of PreCDDP/YC on macrophages was studied. After incubation with various doses of PreCDDP/YC for 24 or 48 h, no significant changes in cell viability occurred (Figure 3C). Even at a dose as high as 500 μg/mL, only slight decrease in cell viability was observed after 48 h of treatment. These data implied that PreCDDP/YC had negligible effect on RAW264.7 cells, at least in the examined dose range.

Also, *in vitro* cell culture experiments were conducted to investigate cytotoxicity of PreCDDP/YC in different tumor cell lines including HepG2 hepatocellular carcinoma cell, HeLa human cervical cancer cell, A549 human lung carcinoma cell, MCF-7 breast cancer cell, and multidrug resistant MCF-7 cell (MCF-7/ADR). First, we found that the empty carrier YC had no cytotoxicity in all tumor cells after incubation for 24 or 48 h at doses varying from 0 to 500 µg/mL (Figure S6). For both PreCDDP and PreCDDP/YC, dose-response cytotoxicity patterns were observed after 24 or 48 h of incubation (Figure S7 and Figure 3D-G), regardless of different cancer cells. Nevertheless, PreCDDP showed stronger cytotoxicity than PreCDDP/YC at the same doses in all the examined CDDP-sensitive tumor cells. This should be attributed to the protective effect of YC in the case of PreCDDP/YC. Interestingly, PreCDDP/YC displayed higher antitumor activity in resistant MCF-7/ADR cells, compared to PreCDDP (Figure 3H and Figure S7E). This might be due to the fact that PreCDDP/YC could decrease the drug efflux from resistant cells through P-glycoprotein and other multidrug resistance-associated proteins, by changing the cellular internalization pathways 66, 72, 73. Consequently, the antitumor activity of CDDP loaded in the form of PreCDDP nanoprecursors in YC was well retained.

### *In vivo* tumor targeting of orally delivered YC in mice bearing A549 xenografts

**Figure 4:**
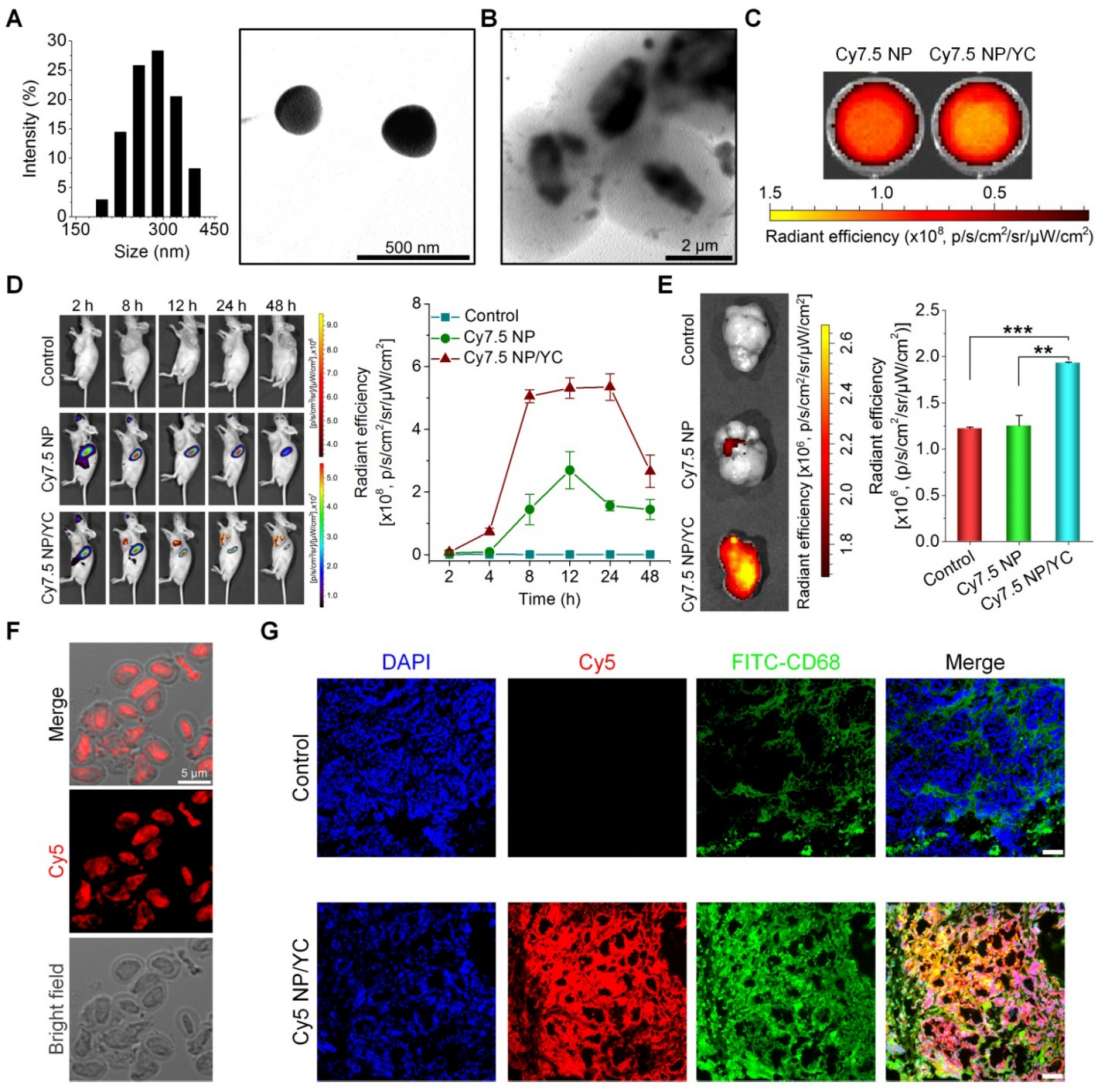
Tumor targeting capability of orally delivered YC in mice with A549 xenografts. (A) Size distribution (left) and TEM image (right) of an assembled near-infrared fluorescent nanoprobe Cy7.5 NP. (B) TEM image of Cy7.5 NP-loaded YC. (C) *Ex vivo* images indicating near-infrared fluorescence of Cy7.5 NP and Cy7.5 NP/YC with excitation at 745 nm and emission at 820 nm. (D) Representative real-time *in vivo* images (left) and quantitative analysis (right) showing *in vivo* tumor targeting capability of Cy7.5 NP/YC. Note that Cy7.5 fluorescent signals were split to clearly show their localization in tumors. (E) *Ex vivo* images (left) and quantification (right) illustrating distribution of Cy7.5 fluorescent signals in isolated A549 xenografts. (F) Microscopic images of Cy5 NP-labeled YC (Cy5 NP/YC). (G) Immunofluorescence images of tumor sections illustrating the co-localization of Cy5 NP/YC with CD68^+^ macrophages. Scale bars in (G) represent 100 μm. Data in (D-E) are mean ± SD (n = 4); **p < 0.01, ***p < 0.001.

We explored *in vivo* tumor targeting performance of orally administered YC in mice. First, a near-infrared fluorescent nanoprobe was constructed by self-assembly of an amphiphile of cyanine 7.5 (Cy7.5)-conjugated PEI, giving rise to a positively charged fluorescent Cy7.5 nanoparticle (Cy7.5 NP) with mean size of ~289 nm and zeta-potential value of +35 mV (Figure 4A). Cy7.5 NP was also packaged into YC by electrostatic force-mediated self-deposition (Figure 4B). At the same dose of Cy7.5, Cy7.5 NP and Cy7.5 NP/YC displayed comparable fluorescent intensities (Figure 4C).

Subsequently, A549 xenografts in mice were established by subcutaneous inoculation of A549 cancer cells. Oral administration of either Cy7.5 NP or Cy7.5 NP/YC was conducted. At 2 h after oral gavage, significant fluorescent signals could be observed in liver and intestinal tissues in mice treated with Cy7.5 NP (Figure 4D, left), which were gradually reduced with time. In this group, only slight fluorescent intensities were found at tumor sites. By contrast, mice administered with Cy7.5 NP/YC showed notably stronger Cy7.5 fluorescent signals in tumors at examined time points, compared to those treated with Cy7.5 NP. Moreover, relatively low fluorescence in the liver and intestine was detected in the Cy7.5 NP/YC group. These findings were affirmed by quantitative analysis (Figure 4D, right). Consistent with *in vivo* results, *ex vivo* imaging of isolated tumors at 48 h revealed remarkably higher fluorescent signals in the Cy7.5 NP/YC group when compared with those of mice administered with Cy7.5 NP (Figure 4E). In this case, however, the Cy7.5 NP group showed no significant difference in comparison to the control group treated with saline.

Furthermore, immunofluorescence analysis was conducted in a separate experiment to explore the distribution of orally delivered YC in the tumor tissue. To this end, Cy5 NP-labeled YC (Cy5 NP/YC) was prepared (Figure 4F), using positively charged Cy5 NP (mean diameter, ~270 nm; zeta-potential, +34 mV) assembled by Cy5-conjugated PEI. Compared with the control group treated with saline, the Cy5 NP/YC group displayed stronger Cy5 signals in the tumor sections of A549 xenografts (Figure 4G). Of note, Cy5 fluorescence was largely co-localized with that due to FITC-labeled CD68 antibody. This result suggested that the tumor targeting effect of orally administered YC was mainly mediated by macrophages.

**Figure 5:**
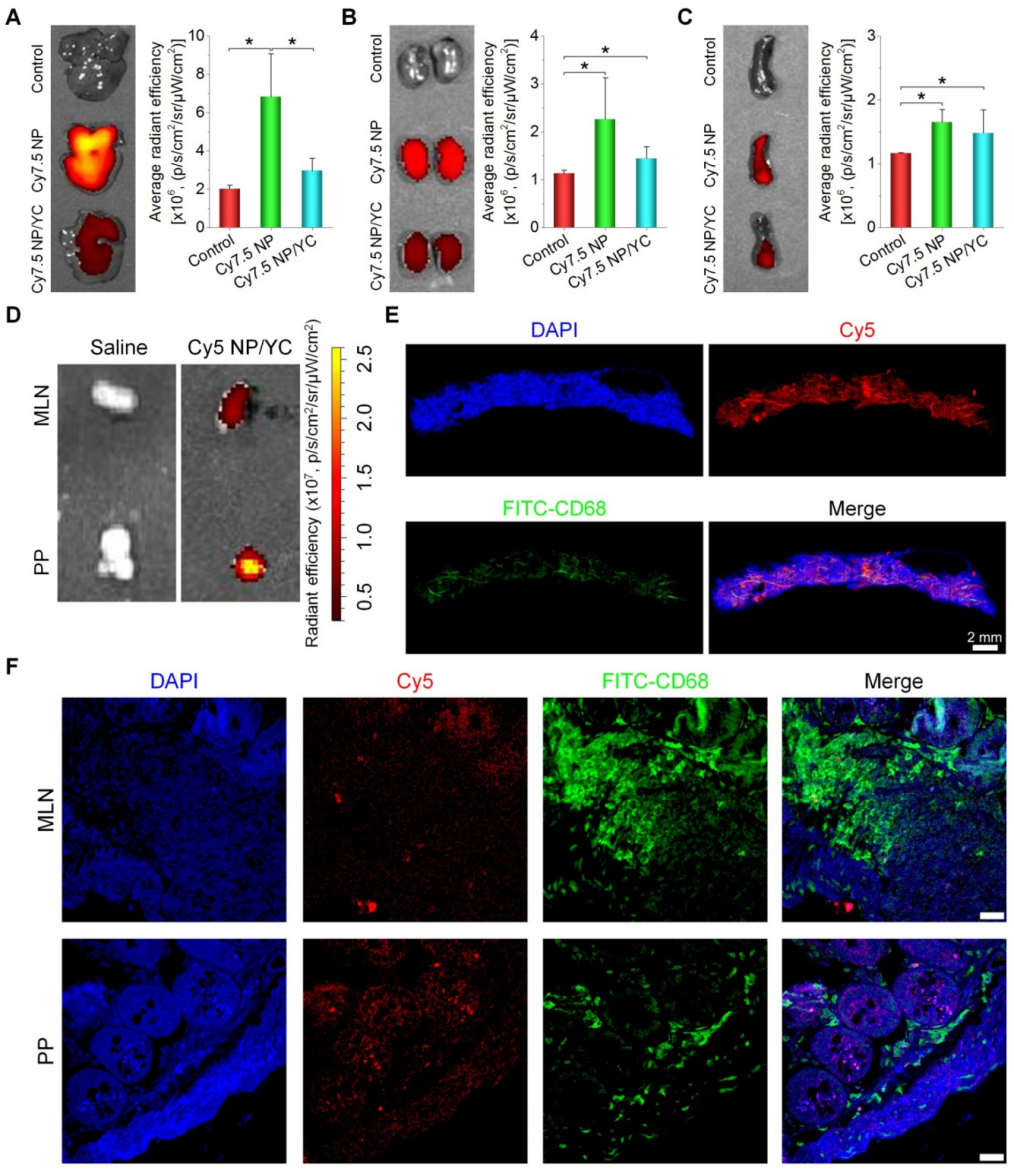
*In vivo* tissue distribution and translocation of orally administered YC in mice with A549 xenografts. (A-C) Typical *ex vivo* images (left) and quantitative data (right) showing distribution of Cy7.5 fluorescent signals in the liver (A), kidney (B), and spleen (C). (D) *Ex vivo* images indicating Cy5 NP/YC in Peyer's patches (PP) and mesenteric lymph nodes (MLN) after oral delivery in mice with A549 xenografts. (E) Whole-slide images of a typical section of MLN isolated from Cy5 NP/YC-treated mice. (F) Confocal microscopy images of MLN and PP sections. Scale bars in (F) represent 200 μm. Data are mean ± SD (n = 4); *p < 0.05.

To further interrogate possible mechanisms underlying tumor targeting capability of orally delivered YC, fluorescence imaging was conducted for organs related to translocation, metabolism, and excretion of microparticles. After oral administration, we found that fluorescent intensities of liver and kidney from Cy7.5 NP-treated mice were notably higher than those of the Cy7.5 NP/YC group (Figure 5A-B), while comparable signals were observed in the spleen (Figure 5C). These data indicated that orally administered Cy7.5 NP was largely transported to the liver and spleen, while it was excreted via the kidney. By contrast,Cy7.5 NP/YC was mainly translocated to the spleen after oral delivery. In separate studies, after oral delivery of Cy5 NP/YC, significant fluorescent signals were observed in both Peyer's patches (PP) and mesenteric lymph nodes (MLN) (Figure 5D), both of which are lymphatic tissues closely related to the intestinal absorption of microparticles 74. Correspondingly, immunofluorescence examination by whole slide imaging of the MLN sections revealed the presence of considerable Cy5 signals (Figure 5E). Moreover, red fluorescence of Cy5 was mainly overlapped with the green fluorescence of CD68^+^ macrophages. Further analysis by confocal microscopy showed the presence of Cy5 signals in both MLN and PP sections (Figure 5F)*,* which were partially co-localized with CD68^+^ macrophages. These results demonstrated that YCs absorbed in the intestine may be dominantly transported via the lymphatic system.

### *In vivo* pharmacokinetic studies

**Figure 6:**
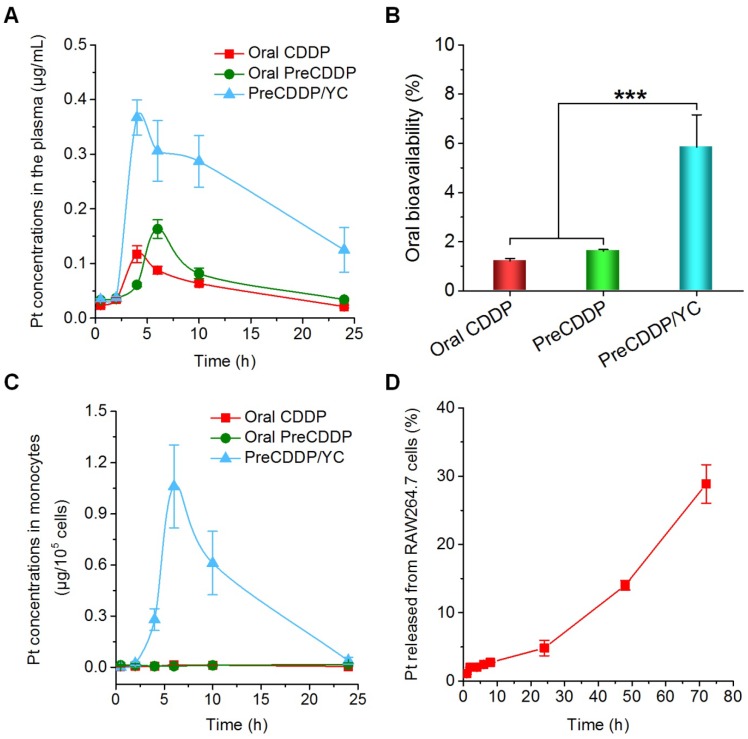
Pharmacokinetic studies after oral administration of different Pt-containing formulations in rats at 6.0 mg/kg. (A) The plasma Pt concentration-time curves after oral delivery of CDDP, PreCDDP, or PreCDDP/YC. (B) Oral bioavailability values of CDDP, PreCDDP, and PreCDDP/YC that were calculated as the AUC ratio of each oral formulation to *i.v.* CDDP. (C) The Pt concentrations in peripheral blood monocytes after oral administration of CDDP, PreCDDP, or PreCDDP/YC. (D) The Pt release profile in RAW264.7 cells after incubation with PreCDDP/YC at 10 μg/mL of PreCDDP for 2 h. Data are mean ± SD (n = 5); ***p < 0.001.

After oral administration of CDDP, PreCDDP, or PreCDDP/YC in rats, the plasma Pt concentration-time curves were determined. At the examined time points post 2 h, the PreCDDP/YC group showed significantly higher Pt levels than those of CDDP and PreCDDP groups (Figure 6A). Further analysis of typical pharmacokinetic parameters indicated that the area under the plasma concentration-time curve (AUC) of PreCDDP/YC was 480% larger than that of oral CDDP (Table 1). Also, the AUC value of PreCDDP/YC was notably higher than that of oral PreCDDP. As a result, the oral bioavailability of CDDP was significantly enhanced by loading its nanoprecursor into YC (Figure 6B). Of note, although *in vitro* tests showed rapid release in the first 6 h (reached an accumulative percentage of 50%), plasma concentrations of Pt were relatively prolonged, which may be due to the different aqueous microenvironments. Under *in vitro* release conditions, a large amount of water were employed as the medium, while there is only a limited amount of water in the gastrointestinal tract.

Our previous studies have demonstrated that orally delivered YCs are mainly distributed in monocytes after they enter the bloodstream, followed by transportation to the diseased sites by monocytes/macrophages 62, 63. Therefore, the Pt concentrations in peripheral blood monocytes were also quantified after oral administration of various Pt formulations. For orally administered PreCDDP and CDDP, extremely low levels of Pt were detected in the isolated monocytes (Figure 6C). By contrast, the PreCDDP/YC group displayed relatively high concentrations of Pt in monocytes. This indicated that after oral administration, PreCDDP/YC was transported to the peripheral blood through the lymphatic system and mainly stored in monocytes. Since the premature release of Pt from peripheral blood monocytes may affect the subsequent transportation to the tumor sites, we examined the Pt release profile in RAW264.7 cells after incubation with PreCDDP/YC (at 10 μg/mL of PreCDDP) for 2 h. Quantification of the Pt concentrations in the culture medium showed slow release of PreCDDP/YC from RAW264.7 cells. After 72 h, only 28.9 ± 2.8 % of total Pt was released into the culture medium (Figure 6D). These results indicated that PreCDDP/YC not only had higher oral bioavailability than CDDP and PreCDDP, but also could be stably stored in monocytes for site-specific delivery to tumors. Upon arrival at the tumor site, the active forms of Pt would be delivered to tumor cells by gradual release of PreCDDP or by transportation via tunneling nanotubes between macrophages and tumor cells 75, 76.

According to the above data, in combination with the fluorescence imaging results, orally delivered PreCDDP/YC was first absorbed by intestinal M cells, and then transported to peripheral blood monocytes/macrophages via the lymphatic system. Because lymph can transfer cells and nutrients to the peripheral blood through the cisterna chyli, this may provide a pathway for the translocation of PreCDDP/YC in lymph to the peripheral blood 77.

### *In vivo* efficacy of orally delivered PreCDDP/YC in mice

**Figure 7:**
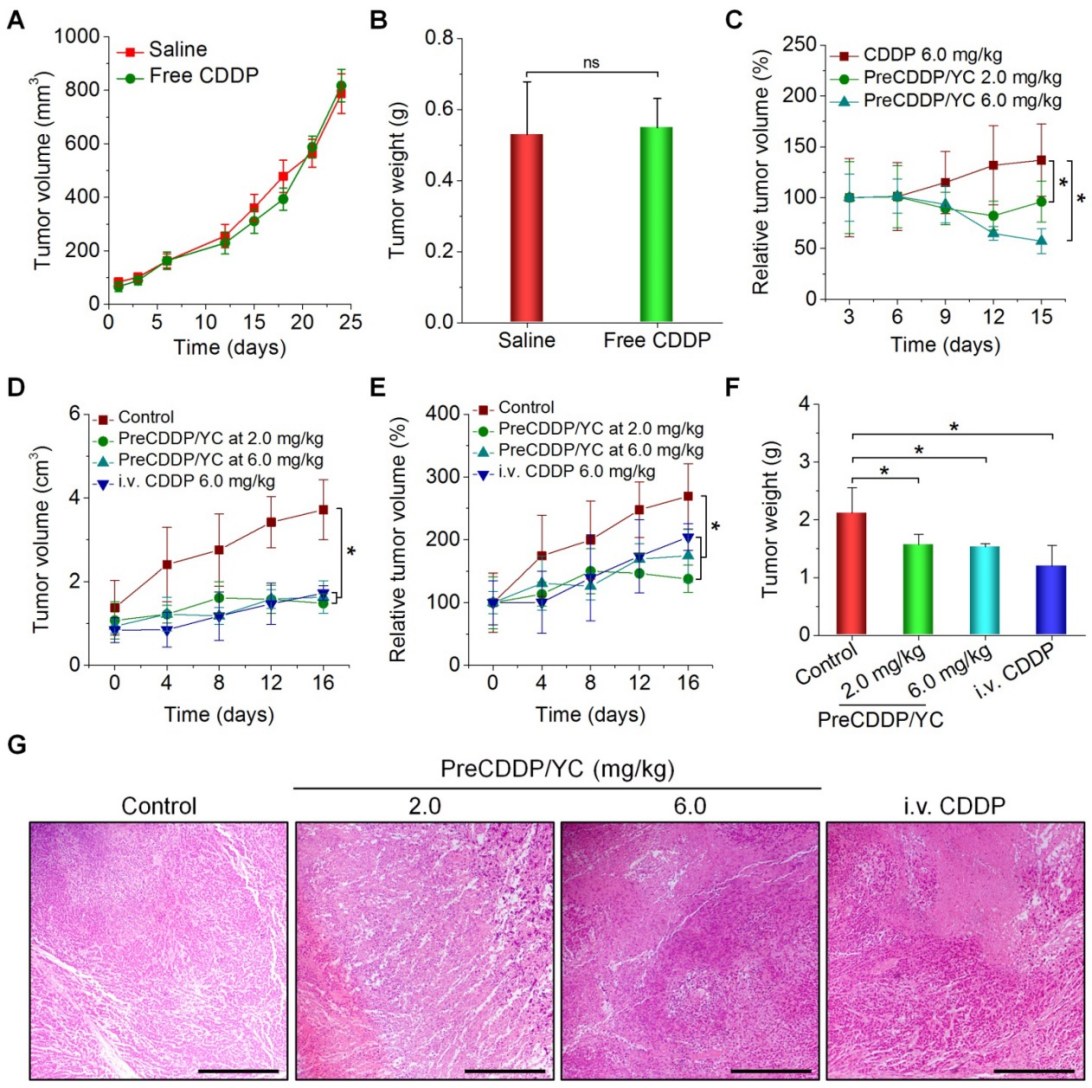
*In vivo* antitumor efficacy of orally administered PreCDDP/YC in mice bearing A549 xenografts. (A) Changes in the tumor volume during treatment with either saline or free CDDP administered by oral gavage. (B) Tumor weight of A549 xenografts at day 24 after treatment. (C) Changes in the relative tumor volume of A549 xenografts during treatment with orally administered free CDDP or PreCDDP/YC. (D-E) The tumor volume (D) and relative tumor volume (E) of A549 xenografts during treatment with orally administered PreCDDP/YC or intravenously injected CDDP (*i.v.* CDDP). Mice in the control group were treated with saline. To calculate the relative tumor volume, measured tumor volumes at varied time points were normalized to those at day 0. (F-G) Tumor weight (F) and H&E-stained sections (G) of A549 xenografts isolated from mice after different treatments. Scale bars, 200 μm. Data are mean ± SD (A-C, n = 6; D-F, n = 8); *p < 0.05; ns, no significance.

Based on the above promising findings, *in vivo* therapeutic effects of PreCDDP/YC were examined in mice bearing A549 xenografts. First, we evaluated efficacies of orally administered free CDDP. After oral administration of CDDP at 6.0 mg/kg every three days in mice, no significant changes in the tumor volume of A549 xenografts were found, compared to the model group treated with saline (Figure 7A). At day 24 post treatment, examination on the weight of isolated tumors also suggested that orally administered CDDP cannot effectively inhibit tumor growth (Figure 7B). This result is consistent with the fact that CDDP must be given to patients by parenteral administration 52, 78. Whereas some studies indicated that CDDP was orally active in certain murine tumors 79, extremely high doses of CDDP were administered each day in the related experiments. In another experiments, *in vivo* antitumor efficacy of orally administered free CDDP and PreCDDP/YC was compared. Whereas mice treated with free CDDP at 6.0 mg/kg showed gradually increased tumor volumes, treatment by PreCDDP/YC at either 2.0 or 6.0 mg/kg of PreCDDP effectively suppressed tumor growth (Figure 7C). This result demonstrated PreCDDP delivered via YC was orally effective. It is worth noting that PreCDDP/YC effectively inhibited tumor growth at either 2.0 or 6.0 mg/kg of PreCDDP without significant difference. This might be due to the fact that PreCDDP/YC was site-specifically delivered to the tumor site by monocytes, while the maximal distribution of PreCDDP/YC in tumors would be limited by the number of migrated monocytes.

Subsequently, we compared the therapeutic performance of orally delivered PreCDDP/YC with CDDP administered via *i.v.* injection. In this cohort of study, PreCDDP/YC was orally administered at 2.0 or 6.0 mg/kg of PreCDDP every three days, while CDDP injection (the clinically used formulation) was given at 6.0 mg/kg via the tail vein. Compared with the saline control, treatment with *i.v.* injected CDDP considerably attenuated tumor growth (Figure 7D-E). Likewise, orally delivered PreCDDP/YC displayed beneficial therapeutic effects at examined doses, which were even comparable to that of *i.v.* CDDP. In addition, detection of isolated tumors revealed that mice treated with different formulations showed significantly smaller tumor weight when compared with that of the model control (Figure 7F), while no significant differences were found between *i.v.* CDDP and oral PreCDDP/YC groups. Further examination on hematoxylin and eosin (H&E)-stained histological sections revealed a considerable number of necrotic and apoptotic cells for all CDDP-treated groups, clearly different from that treated with saline (Figure 7G). Taken together, our results demonstrated that PreCDDP orally delivered via YC exhibited desirable antitumor efficacy, which was even comparable to that of *i.v.* administered CDDP in the examined A549 tumor model.

### Preliminary safety studies in mice

**Figure 8:**
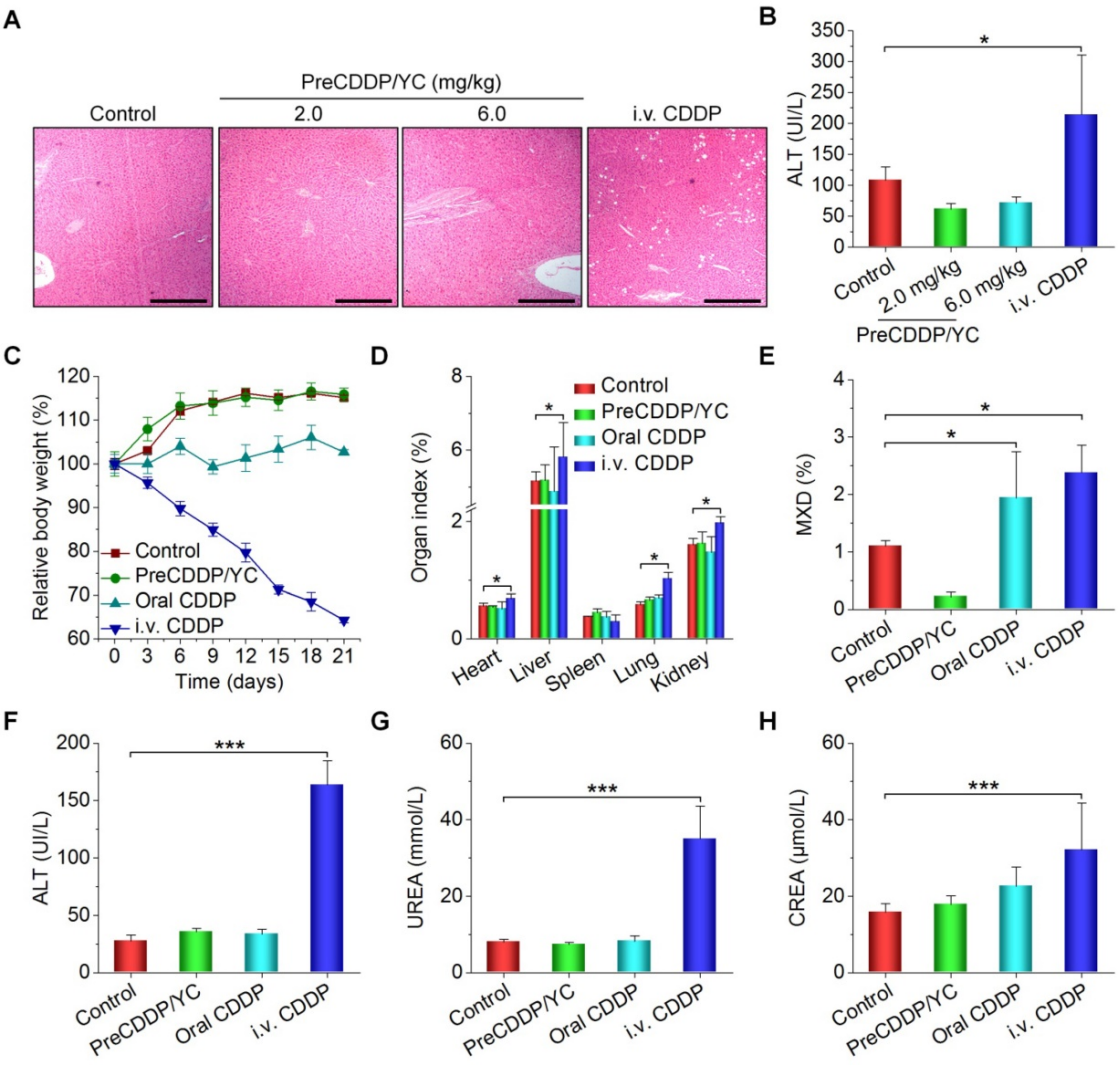
*In vivo* safety study of different CDDP formulations in mice. (A-B) H&E-stained sections of liver isolated from A549 xenograft-bearing nude mice (A) and the serum ALT level (B) after treatment with either orally administered PreCDDP/YC or intravenously injected CDDP at 6.0 mg/kg. Scale bars, 200 μm. (C) Changes in the relative body weight of BALB/c mice during treatment with different formulations. (D-H) The organ index (D), the mixed cell count percentage (MXD) (E), as well as the levels of ALT (F), UREA (G), and CREA (H) after different treatments. Mice in the PreCDDP/YC and Oral CDDP groups were treated every three days by oral administration at a CDDP dose of 6.0 mg/kg, while the *i.v.* CDDP group was intravenously injected with the same dose of CDDP every three days. In all cases, mice in the control group received saline by oral gavage. Data are mean ± SD (B, n = 8; C-H, n = 6); *p < 0.05, ***p < 0.001.

Finally we evaluated *in vivo* safety profiles of orally deliverable PreCDDP/YC. After treatment of nude mice with different CDDP formulations, we found significant and numerous vacuoles in H&E-stained sections of the liver excised from the *i.v.* CDDP group (Figure 8A). By contrast, only less and very small vacuoles appeared in the PreCDDP/YC group at the same dose of 6.0 mg/kg. At a low dose of 2.0 mg/kg, normal microstructure was observed in the H&E section corresponding to the PreCDDP/YC group. The vacuole structure should be related to hepatic macrovesicular steatosis. Consistently, a notably high level of alanine aminotransferase (ALT) was detected for the *i.v.* CDDP group (Figure 8B). This CDDP-induced liver toxicity was also reported previously in both clinical and animal studies 80-82. On the other hand, histopathological evaluation on H&E-stained sections of intestinal tissues revealed that oral administration of CDDP caused evident irritation and inflammatory infiltration at the proximal and distal segments of intestinal tissues (Figure S8). In contrast, oral delivery of PreCDDP/YC did not lead to significant injuries in the whole intestinal tissues.

Then we performed systemic safety studies in BALB/c mice. To this end, different CDDP formulations were administered at 6.0 mg/kg CDDP every three days. During treatment, mice in the PreCDDP/YC group exhibited gradual body weight gain comparable to that of the control group administered with saline (Figure 8C). While animals orally administered with free CDDP showed relatively low weight increases, body weight of mice in the *i.v.* CDDP group notably decreased. After 6 times of consecutive administration, mice were euthanized. Compared with the control group, the *i.v.* CDDP group showed significantly increased organ index for heart, liver, lung, and kidney (Figure 8D), indicating the existence of swelling in these examined major organs. Also, we observed the notably increased mixed cell count percentage (MXD) of monocytes, basophils, and eosinophils in both orally and *i.v.* administered CDDP groups (Figure 8E).

**Figure 9:**
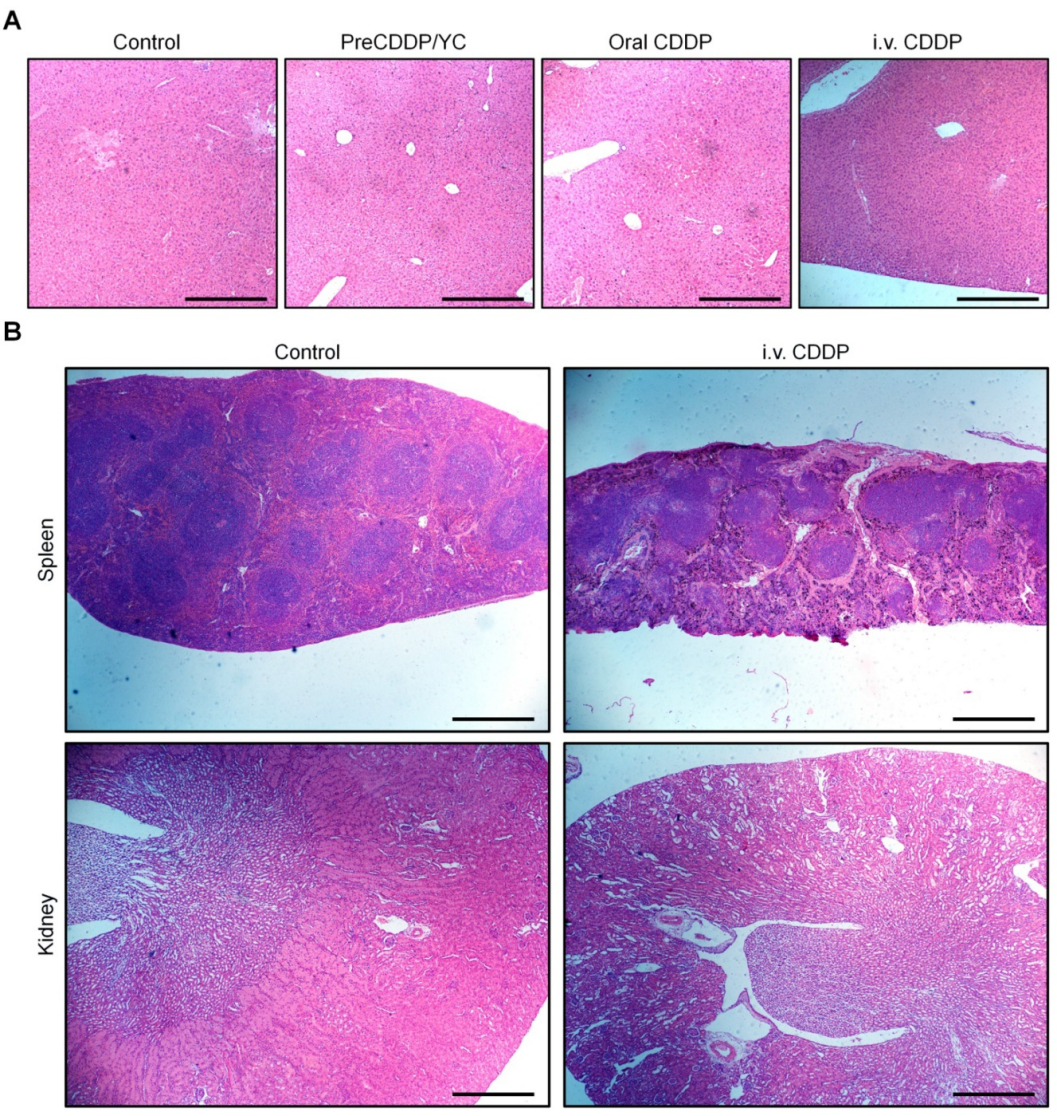
H&E-stained pathological sections of representative organs after treatment with different formulations. (A) Liver. (B) Spleen and kidney. Different organs were resected from BALB/c mice after three weeks of treatment. Scale bars, 200 μm (A), 500 μm (B).

Additional clinical biochemical tests revealed dramatically increased levels of ALT, blood urea (UREA), and creatinine (CREA) for mice in the *i.v.* CDDP group (Figure 8F-H). These data suggested that abnormal changes in both hepatic and kidney functions occurred. Correspondingly, we found significant infiltration of inflamed cells in the H&E-stained liver section of *i.v.* CDDP-treated mice (Figure 9A). Furthermore, considerable numbers of necrotic or apoptotic cells could be clearly observed in spleen and kidney sections of the *i.v.* CDDP group (Figure 9B). While liver toxicity has been reported in some cases after chemotherapy with CDDP, dose-limiting nephrotoxicity is a well-recognized adverse effect related to CDDP treatment 2, 83, 84. Collectively, these results demonstrated that, at the examined dose and dosing regimen, free CDDP administered via the oral or *i.v.* route displayed side effects to varied degrees in both major organs and intestinal tissues, particularly in the case of *i.v.* CDDP. Comparatively, orally delivered PreCDDP/YC showed good safety profiles, which should be largely due to the protective effect of YC on PreCDDP, the special intestinal absorption pathway of YC, and the low distribution of YC in the liver, spleen, and kidneys. Moreover, YC itself is a safe drug carrier for oral delivery, in view of the fact that specific preparations of yeast-derived β-glucan have received Generally Recognized as Safe status and acceptance as novel food ingredients by European Food Safety Authority 85. Nevertheless, further comprehensive *in vivo* evaluations are necessary to address the potential immunotoxicity of both YC and PreCDDP/YC, particularly after long-term treatment.

## Conclusions

In summary, herein we established the optimal parameters relevant to alkali and acidic treatments of yeast cells to fabricate YCs with improved loading capability. Using a visible fluorescent QD, desirable packaging capacity of YC was substantiated. *In vitro* cell culture experiments demonstrated that YC can be rapidly endocytosed by macrophages, while the internalized YC can be retained in cells for a relatively long period of time. On the other hand, CDDP was efficiently encapsulated in YC by transforming into a water-soluble nanoprecursor PreCDDP, which can be effectively released in buffers simulating gastrointestinal conditions. Thus formulated PreCDDP/YC showed potent antitumor activity in different tumor cells, indicating that the loaded PreCDDP remained active after intracellular release. Orally administered YCs could accumulate at the tumor sites of A549 xenografts in mice, realizing by monocyte/macrophage-mediated translocation via the lymphatic system. Based on this targeting effect, oral delivery of PreCDDP/YC showed desirable therapeutic benefits in mice bearing A549 xenografts, which were comparable to that of the same dose of free CDDP administered by *i.v.* injection. By contrast, oral gavage of free CDDP did not afford antitumor efficacy. Furthermore, preliminary studies revealed that oral treatment with PreCDDP/YC displayed good safety profiles as compared to free CDDP administered via the oral or *i.v.* route. These findings suggested that this YC-mediated oral delivery approach is an effective biomimetic strategy to develop oral active chemotherapies from CDDP or its derivatives.

## Figures and Tables

**Table 1:** Representative pharmacokinetic parameters of different formulations.

Formulations	*AUC*_(0-∞)_ (μg/mL*h)	*C*_max_ (μg/mL)	*t*_1/2_ (h)
*i.v.* CDDP	128.9 ± 2.4	18.5 ± 1.0	2.2 ± 0.1
Oral CDDP	1.6 ± 0.1	0.1 ± 0.02	8.8 ± 0.5
Oral PreCDDP	2.1 ± 0.1	0.2 ± 0.01	8.7 ± 0.7
PreCDDP/YC	6.7 ± 0.5^*,#^	0.4 ± 0.04	10.0 ± 0.1^*,#^

^*^p < 0.05 versus oral CDDP, ^#^p < 0.05 versus oral PreCDDP.
