# Supplementary material for: Targeted Delivery of Cisplatin-Derived Nanoprecursors via a Biomimetic Yeast Microcapsule for Tumor Therapy by the Oral Route
Source: Theranostics. 2019 Aug 21;9(22):6568–86. doi: 10.7150/thno.35353 (PMC6771252; doi:10.7150/thno.35353)
Supplement: Supplementary file 1 — Supplementary figures and tables. [file thnov09p6568s1.pdf]

Supplementary materials

Targeted Delivery of Cisplatin-Derived Nanoprecursors via a Biomimetic Yeast Microcapsule for Tumor Therapy by the Oral Route

Xing Zhou[1,*], Kaijian Ling[2,*], Mengyu Liu[1,*], Xiangjun Zhang[1], Jun Ding[3], Yan Dong[4], Zhiqing Liang[2], Jianjun Li[4], Jianxiang Zhang[1]

1. Department of Pharmaceutics, College of Pharmacy, Third Military Medical University, Chongqing 400038, China
2. Department of Obstetrics and Gynaecology, Southwest Hospital, Third Military Medical University, Chongqing 400038, China
3. Department of Ultrasound, Southwest Hospital, Third Military Medical University, Chongqing 400038, China
4. Department of Oncology and Southwest Cancer Center, Southwest Hospital, Third Military Medical University, Chongqing 400038, China

[*]These authors contributed equally to this work.

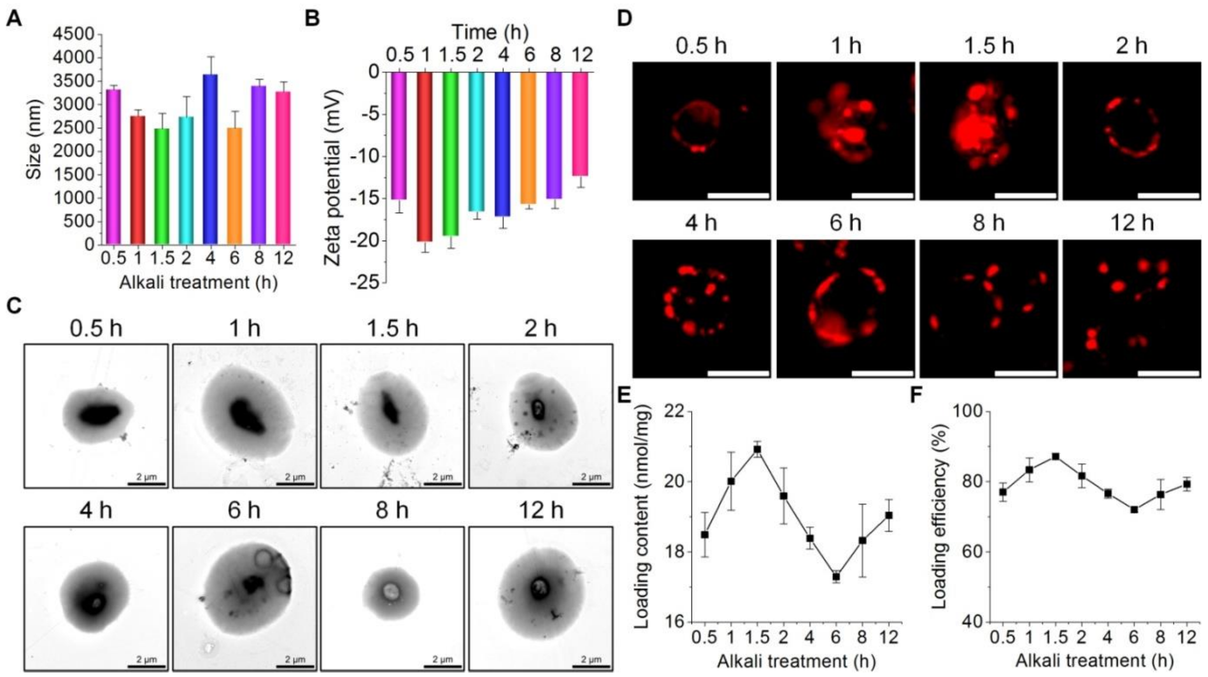

Figure S1. The effects of alkali treatment time on physicochemical properties and loading capacity of YCs. (A-C) Average size (A), zeta-potential values (B), and TEM images (C) of YCs obtained after treatment with alkali for different periods of time. (D) Representative CLSM images of QD620-loaded YCs based on YCs obtained after alkali treatment for various time periods. (E-F) The effect of alkali treatment time on QD620 loading contents (E) and loading efficiency (F) of YCs. The scale bars in (D) represent 4 μm. Data are mean ± SD (n = 3). For all YCs, the acid treatment time was 1 h.

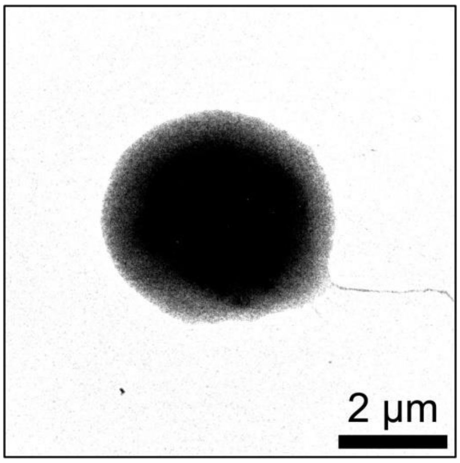

Figure S2. A representative TEM image showing an intact yeast cell.

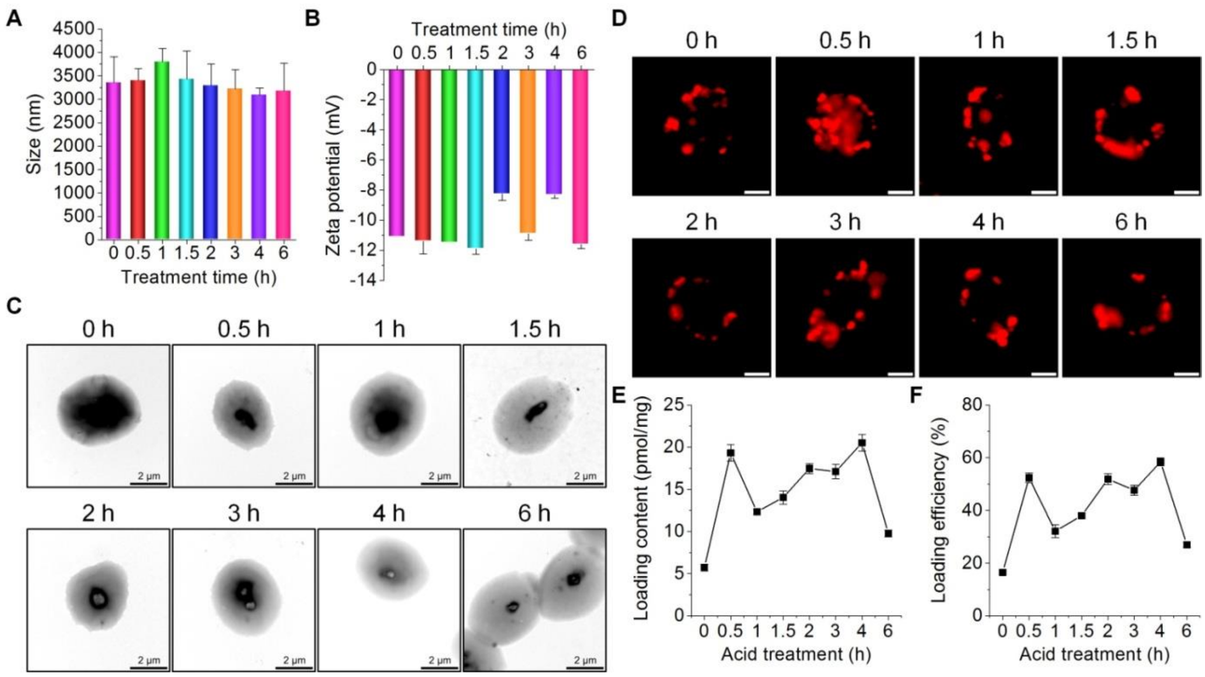

Figure S3. The effects of acid treatment time on physicochemical properties and loading capacity of YCs. (A-C) Average size (A), zeta-potential values (B), and TEM images (C) of YCs obtained after acidic treatment for different periods of time. (D) Representative CLSM images of QD620-loaded YCs derived from YCs prepared with acid treatment for various time periods. (E-F) The effects of acid treatment time on QD620 loading efficiency (E) and loading contents (F) of YCs. The scale bars in (D) represent 2 μm. Data are mean ± SD (n = 3). For all YCs, the alkali treatment time was 1 h.

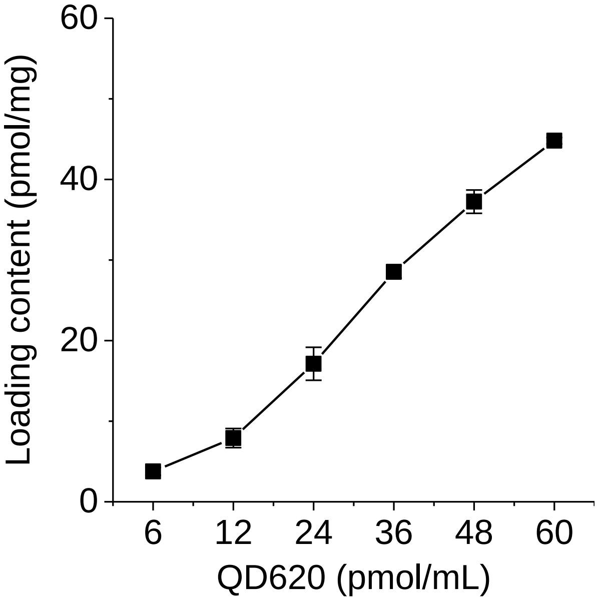

Figure S4. The effect of QD620 feeding on its loading content in YC.

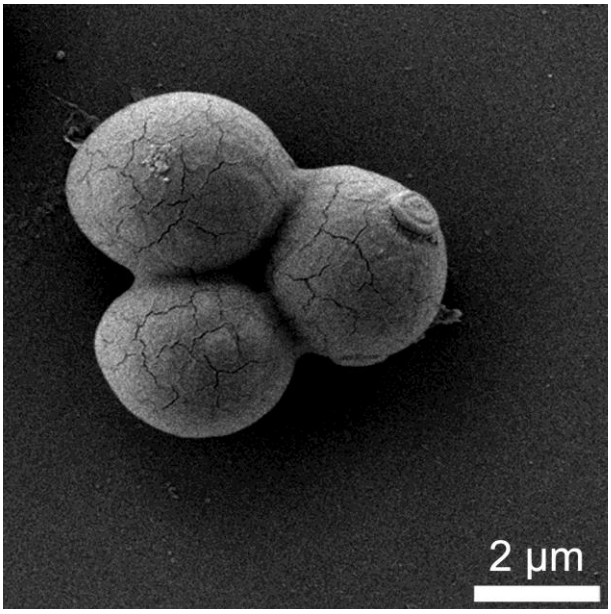

Figure S5. A typical SEM image showing intact yeast cells before removal of core contents.

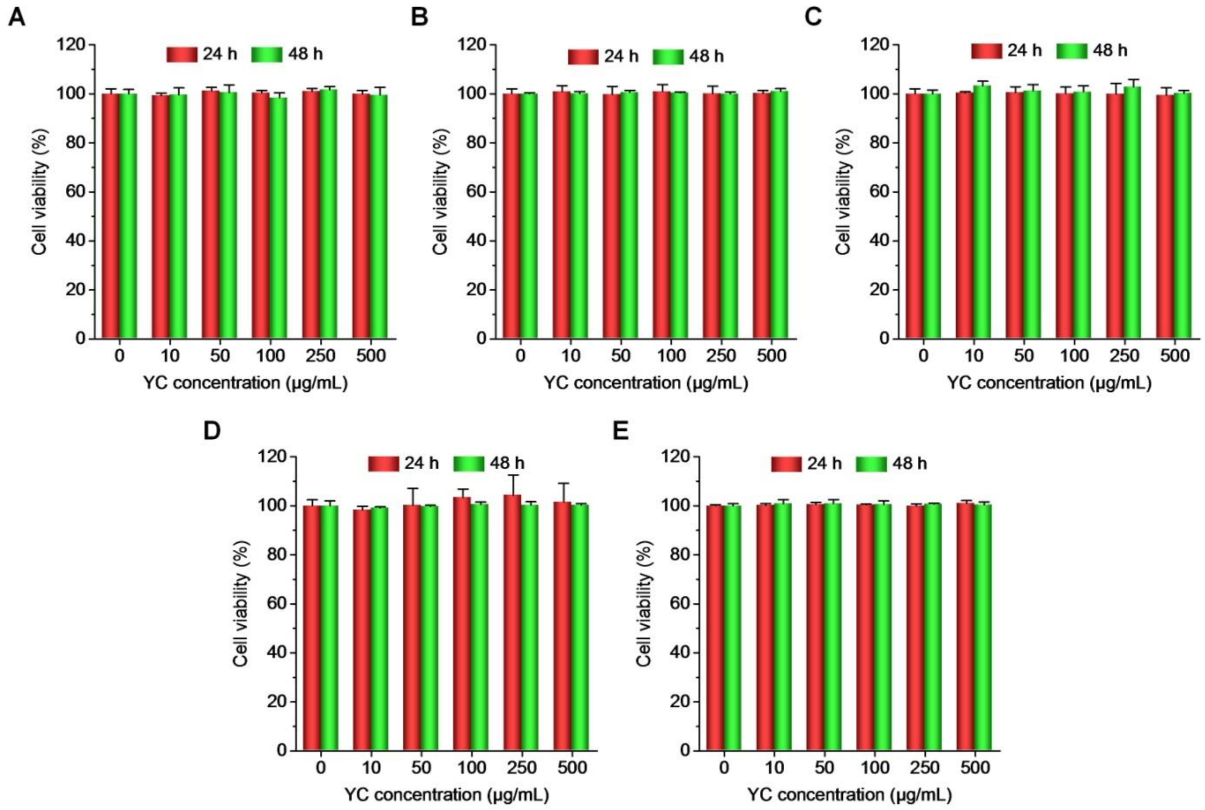

Figure S6. Cytotoxicity of YC in different tumor cells after incubation for 24 or 48 h. (A-E) HepG2 hepatocellular carcinoma cell (A), HeLa human cervical cancer cell (B), A549 human lung carcinoma cell (C), MCF-7 breast cancer cell (D), and multidrug resistant MCF-7 cell (MCF-7/ADR) (E). Data are mean ± SD (n = 5).

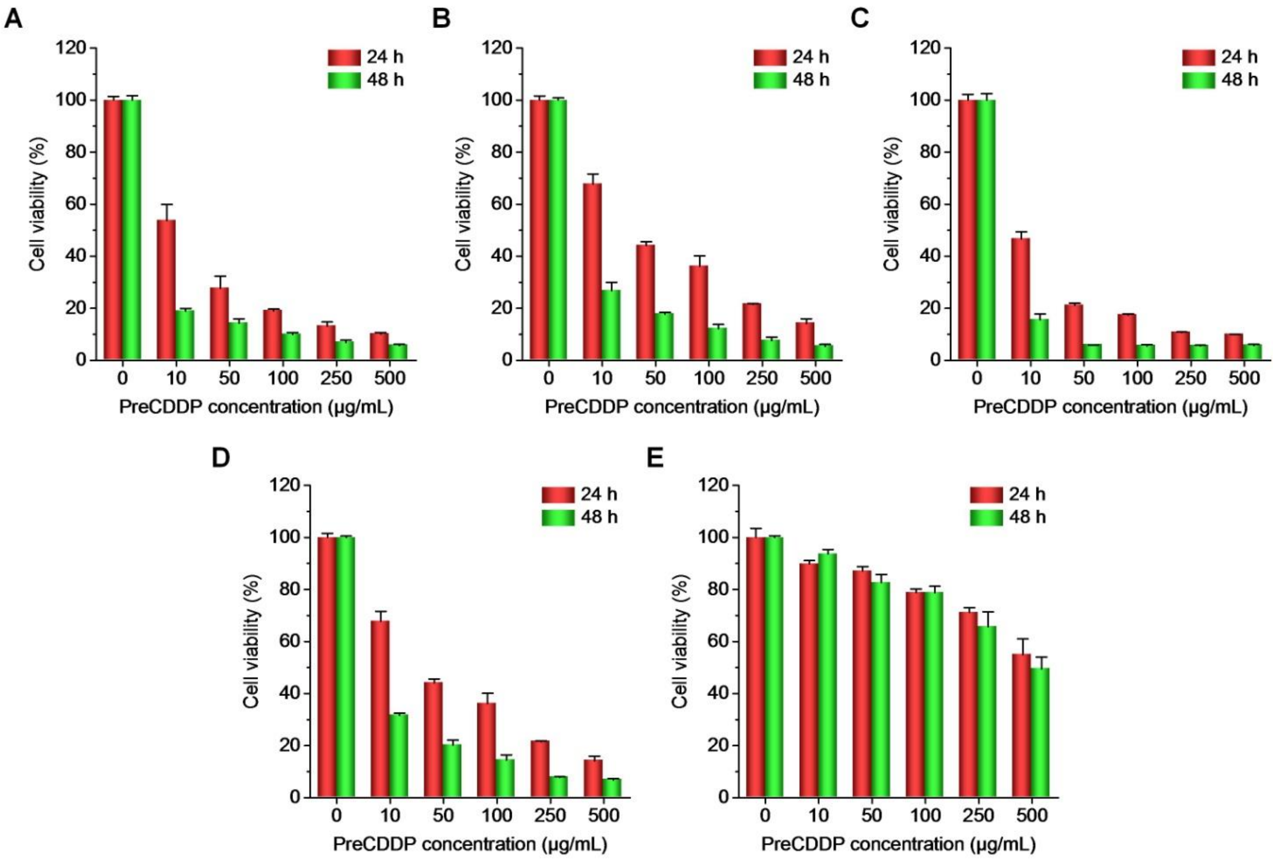

Figure S7. Cytotoxicity of PreCDDP in different tumor cells after incubation for 24 or 48 h. (A-E) HepG2 hepatocellular carcinoma cell (A), HeLa human cervical cancer cell (B), A549 human lung carcinoma cell (C), MCF-7 breast cancer cell (D), and multidrug resistant MCF-7 cell (MCF-7/ADR) (E). For all cell viability experiments, the indicated concentrations represent the doses of PreCDDP. Data are mean ± SD (n = 5).

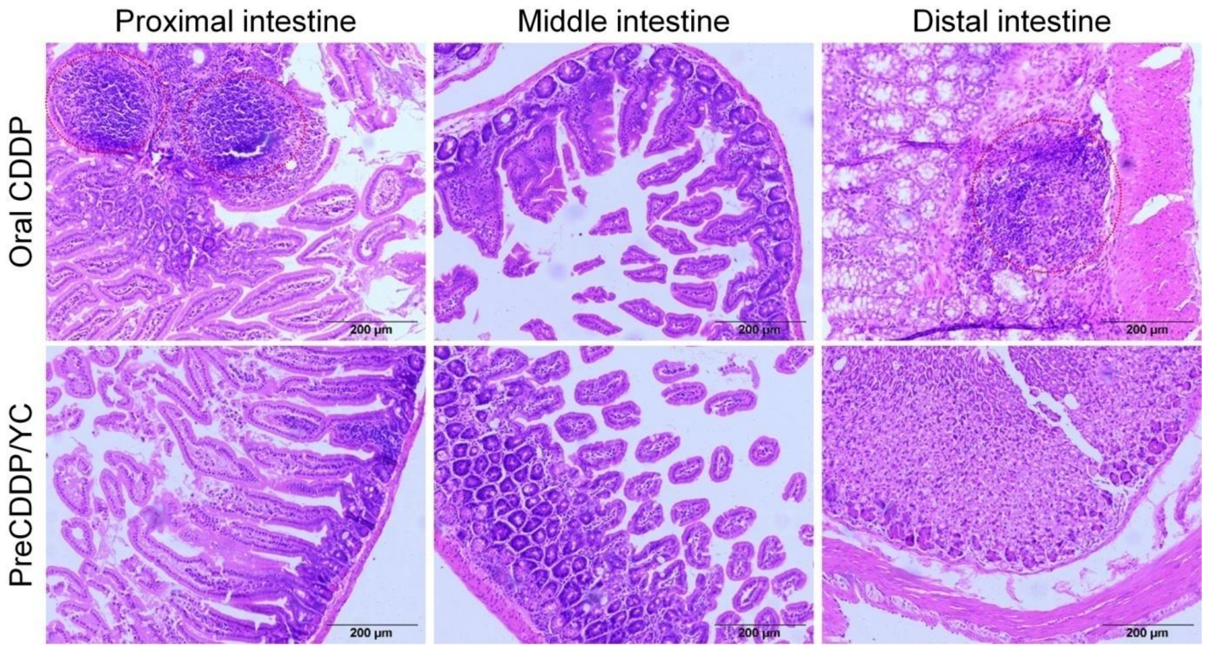

Figure S8. H&E-stained histopathological sections of intestinal tissues from nude mice subjected to various treatments. The red circles indicate inflammatory infiltration.
